# Numerical Characterization of Piezoceramics Using Resonance Curves

**DOI:** 10.3390/ma9020071

**Published:** 2016-01-27

**Authors:** Nicolás Pérez, Flávio Buiochi, Marco Aurélio Brizzotti Andrade, Julio Cezar Adamowski

**Affiliations:** 1 Grupo de Ingeniería Aplicada a los Procesos Agrícolas y Biológicos, Centro Universitario de Paysandú, Universidad de la República, Ruta 3, Km 363, 60000 Paysandú, Uruguay; 2 Departamento de Engenharia Mecatrônica e de Sistemas Mecânicos, Universidade de São Paulo, Avenida Professor Mello Moraes 2231, CP 05508-030 São Paulo, Brazil; fbuiochi@usp.br (F.B.); jcadamow@usp.br (J.C.A.); 3 Instituto de Física, Universidade de São Paulo, CP 05508-090 São Paulo, Brazil; marcobrizzotti@gmail.com

**Keywords:** piezoelectric ceramic, complex parameters, optimization, finite elements

## Abstract

Piezoelectric materials characterization is a challenging problem involving physical concepts, electrical and mechanical measurements and numerical optimization techniques. Piezoelectric ceramics such as Lead Zirconate Titanate (PZT) belong to the 6 mm symmetry class, which requires five elastic, three piezoelectric and two dielectric constants to fully represent the material properties. If losses are considered, the material properties can be represented by complex numbers. In this case, 20 independent material constants are required to obtain the full model. Several numerical methods have been used to adjust the theoretical models to the experimental results. The continuous improvement of the computer processing ability has allowed the use of a specific numerical method, the Finite Element Method (FEM), to iteratively solve the problem of finding the piezoelectric constants. This review presents the recent advances in the numerical characterization of 6 mm piezoelectric materials from experimental electrical impedance curves. The basic strategy consists in measuring the electrical impedance curve of a piezoelectric disk, and then combining the Finite Element Method with an iterative algorithm to find a set of material properties that minimizes the difference between the numerical impedance curve and the experimental one. Different methods to validate the results are also discussed. Examples of characterization of some common piezoelectric ceramics are presented to show the practical application of the described methods.

## 1. Introduction

Piezoelectric ceramics can be found in a great variety of electronic and electromechanical devices. Applications range from cell phones to medical imaging ultrasound, ignition systems to energy harvesting or from motors and actuators to power ultrasound systems for chemical processing.

The use of CAD tools for designing electromechanical systems based on piezoelectric ceramics is a common practice in the industry. The behavior of such systems can be reproduced with a high degree of confidence by using the Finite Element Method (FEM) or similar numerical tools. However, the modeling accuracy is limited by the knowledge of the material properties. In the case of homogenous and isotropic materials such as metals, plastics or conventional ceramics, two independent elastic constants are needed. However, more complex engineering materials are anisotropic and require large number of elastic parameters to fully represent their behavior. As an example, one-directional carbon fiber composites use five independent elastic constants. The case of piezoelectric materials is more complex, because depending on the symmetry of the material, up to 21 elastic, 18 piezoelectric and 6 dielectric independent constants can be found.

**Figure 1:**
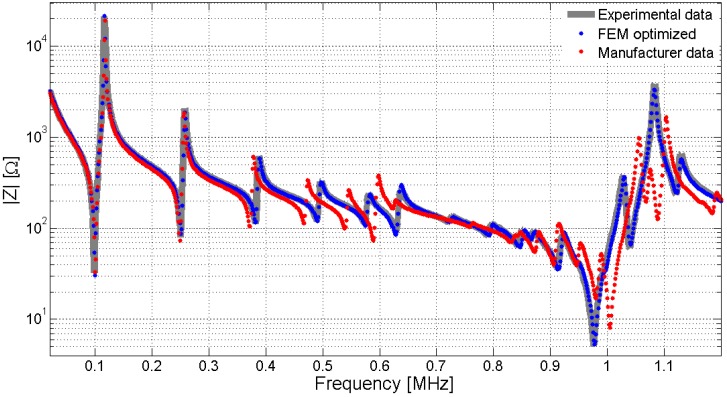
Simulated and experimental impedance of a Pz27 ceramic, 20 mm diameter and 2 mm thickness. The gray continuous line is the experimental impedance, dotted red is the Finite Element Method (FEM) simulation using manufacturer data and dotted blue is the FEM simulation using optimized data. The optimized data is obtained as the mean value of the properties for sixteen samples with different radius and thickness.

This review presents a methodology to identify the parameters in the piezoelectric model based on the minimization of the difference between the experimental and the FEM simulations. In this methodology, the FEM results predict the electrical impedance or the mechanical displacement, and then an objective function measuring the error from the experimental data is minimized by using an optimization algorithm. The procedure is implemented through a loop that runs until an exit condition is achieved. This methodology allows a very precise fitting of the experimental data. The theory and the examples presented here are limited to the most common type of piezoelectric materials used in the fabrication of ultrasonic transducers, the 6 mm symmetry class piezoelectric ceramics. However, the methodology can be extended to more complex materials, as long as these are correctly described by the material constitutive equations. Figure 1 shows the comparison between the experimental data and the FEM simulation using the manufacturer data and the optimized data. The comparison is performed over a disk (20 mm diameter and 2 mm thickness) of soft piezoelectric ceramic Pz27 (Ferroperm Piezoceramics A/S, Kvistgard, Denmark) [1].

In Figure 1, there exists a great discrepancy between the results from the properties supplied by the manufacturer and by using the optimized properties. Some questions must be answered here. First, why do the manufacturer data present large discrepancy? There are two main causes. The manufacturers have a tolerance in the fabrication process, but even for the same material, same geometry and same fabrication batch, the material properties present a statistical dispersion. In addition, manufacturers apply the traditional methodology based on one-dimensional models, using samples with different geometries to determine the properties, thus increasing the dispersion in the results. The major source of dispersion is the inconsistence in the properties obtained from different samples and not the measurement procedure itself. This lack of consistency can be avoided by characterizing the properties using only one sample. 

The second question is: What is the applicability of the data usually supplied by the manufacturers? In most applications, piezoelectric ceramics work in the thickness mode or in the first radial mode. The behavior of these modes is usually well reproduced by the data supplied by the manufacturer. 

The final question is: What is the practical sense of a methodology for precisely determining the full set of material properties? Here, we can distinguish between the manufacturer and the final user point of view. For the manufacturer, the statistical analysis of the data obtained can be used for quality control to determine the differences in the properties introduced by the fabrication process. The statistical dispersion of the properties can be strongly reduced by characterizing samples of the same geometry. From the point of view of the final user, one has a specific sample and, from several applications, it makes sense to obtain an optimized set of properties that reproduces the behavior of this specific sample. This specific set of parameters can be obtained by users themselves or it could be supplied by the manufacturer as a calibration datasheet, in the same manner as for hydrophones and transducers. This service has been unavailable so far, but it could be easily implemented by introducing the methodology described here.

The FEM is widely used to simulate mechanical structures and electromagnetic phenomena. The application of FEM to piezoelectric structures started in the late sixties with the work by Allik and Kagawa [2,3]. These models were developed to simulate harmonic, modal and time analysis [4,5,6]. In a brief review made by Benjeddou back in 2000, more than one hundred papers describing the use of FEM modeling in piezoelectric structures were cited [7]. 

Using FEM modeling, the properties of a piezoelectric ceramic can be identified by minimizing the difference between the numerical and the experimental impedance data. The application of this technique is recent, and the first references are from about fifteen years ago [8,9,10,11]. A comprehensive review of this technique to optimize mechanical structures can be found in the book of Friswell and Mottershead [12]. 

All FEM solutions based on optimizations require an initial condition to start the minimization and a nonlinear minimization technique to obtain the parameters. Although the determination of the impedance can be formulated as a system of partial differential equations, the inverse determination of parameters from the impedance data is a highly nonlinear and ill-conditioned problem. Several nonlinear techniques are proposed to solve this inverse problem. Researchers of the Department of Sensor Technology at the German Friedrich-Alexander University make important contributions to the application of Newton-conjugate gradient and regularization techniques [13,14,15]. Usually, the experimental data is the electrical impedance. However, in FEM simulations, the results can also be nodal displacement or nodal velocity. Ruspich and Lerch presented the simultaneous use of electric and velocity data as input data in the optimization algorithm [15]. The model is solved using complex parameters, and then the origin of the energy losses can be directly associated with the elastic, dielectric or piezoelectric sources. Another classic technique to solve nonlinear minimization problems is the Nelder-Mead method [16]. Andrade and co-authors proposed the use of this optimization algorithm to obtain the material properties of a piezoelectric disk [17]. Following this work, Perez and co-authors proposed the implementation of a physical-based algorithm to obtain the initial conditions for the optimization process [18,19]. This methodology was applied to several examples including rings and disks [20,21,22,23]. 

The FEM identification of the piezoelectric model is under investigation. Several authors have investigated improvements in the determination of the less sensitive parameters. Unverzagt and co-authors analyzed the electrode shape [24,25] and Lahmer and co-authors determined the optimal frequency set to make the inverse algorithm more robust [26]. Another work using the fusion of acoustic and impendence data, presented by Li and co-authors [27], shows a method to determine the elastic constants from acoustic pulse-echo measurements reducing the number of constants to be identified. This methodology can be applied to samples with high degree of anisotropy [28]. Jonsson and co-authors used a wide frequency range including the third harmonic overtone for adjusting the model. They also classified the constants by the frequency range in which they are more sensitive [29]. Another gradient-based algorithm uses the Method of Moving Asymptotes (MMA) [30]. Recently, Kiyono and co-authors used this method to obtain the full piezoelectric model [31,32]. In all the FEM iterative methods the reduction of the model to minimize the simulation time is critical. In a recent work, Rupitsch and Ilg extended the optimization methodology to reach non-axisymmetric samples by dividing the frequency spectrum in zones dominated by modes that can be solved in 2D and 3D [33]. This work also addresses the dependence of the parameters on the temperature. This important fact was also studied by Tang and Cao for PZT-4 ceramics commonly used in power ultrasound applications [34].

The aim of the present work is to review the concepts of the characterization of piezoelectric ceramics using FEM optimization. The scope is restricted to axisymmetric samples belonging to the 6 mm symmetry class with the polarization axis along the thickness. The characterization of piezoelectric ceramics involves some simplifying assumptions: The material is assumed homogenous and isotropic, without cracks of defects in the body [35,36,37,38]. The parameters are considered independent of temperature and frequency, and the geometric model of the sample is assumed as a perfect cylinder.

To give a complete view, Section 2 presents the constitutive equations, introducing the Voigt notation and the reduction of the number of independent parameters in the 6 mm symmetry case. Section 3 presents some basic definitions for the characterization of piezoelectric ceramics using impedance curves and one-dimensional models. Some phenomenological parameters usually given by manufacturers are also explained. In Section 4, the piezoelectric FEM modelling is briefly described. In Section 5, the optimization technique is described. This section is divided into three parts. First, the basic statements for this optimization problem are given. Second, the importance of the initial conditions is highlighted. To show the dependence of the solution on the initial conditions, a sensitivity analysis is presented. Third, some nonlinear optimization strategies to minimize the objective function are reviewed. Finally, Section 6 also presents some strategies to validate the results.

## 2. Piezoelectric Materials and Constitutive Equations

In piezoelectric materials, mechanical and electrical properties are linked, *i.e*., an external electric field can produce a mechanical deformation and, reciprocally, a mechanical stress produces an electric field inside the material or a voltage between the external faces. In low external electric fields, deformation and electric field inside the material are proportional. When the material is subjected to a mechanical strain, the internal distribution of charges changes and produces an electrical polarization, resulting in an electric field proportional to the applied strain. This behavior is named direct piezoelectric effect. On the other hand, if an external electric field is applied, a mechanical strain appears in the same material. This behavior is often named inverse piezoelectric effect. 

The piezoelectric effect was discovered by Jacques and Pierre Curie in the late 19th century [39]. The earlier experiments were made using crystals, such as quartz and Rochelle salt. In the beginning, the piezoelectric effect was associated with the crystalline structure and the symmetry class of the crystal [40]. In the interwar period, the research for more efficient piezoelectric transducers led to the discovery of piezoelectricity associated with ceramic materials. The interested reader is referred to the classic book by Jaffe and co-authors [41]. Citing the authors, “*The term piezoelectric ceramics would have appeared as a contradiction in itself to a physicist as late as 1944*”. At the moment, a number of practical piezoelectric devices are made using piezoelectric ceramics, whereas the crystals are still widely used in electronic oscillators. 

Piezoelectric ceramics have different properties depending on the precise chemical composition, the quality of the chemical components, the sinterization process and the polarization, just to name a few. For that reason, a great dispersion exists between the values of the piezoelectric parameters even for the same manufacturer and the same fabrication batch. If a precise characterization of a piezoelectric ceramic is required, it must be made on the desired sample itself. The use of samples from different fabrication batches to characterize the properties can introduce high dispersions in the results.

The mechanical behavior is described by two tensorial magnitudes, strain tensor *S_i,j_* and stress tensor *T_i,j_*. Both are symmetric tensors, allowing the reduction of the nine component tensor to a six-dimensional vector representation, *S_p_* and *T_p_*. This vector representation is also named Voigt notation. All the parameters obtained in this paper are expressed using the conventions and the notation defined in the IEEE standard [42]. However, the reader must be aware that some types of FEM software use a different representation for the reduced vectors. Moreover, some papers and technical manuals use the notation (*x*, *y*, *z*) to indicate the directions (1, 2, 3). Table 1 presents the equivalence between tensorial components and reduced notation. The convention adopted in the FEM software ANSYS (Ansys Inc., Concord, MA, USA) [43] is also included as an example of a different representation. 

**Table 1:** Relationship between tensor and vector notation (vector index) in IEEE and ANSYS convention.

Tensor Number	Tensor Letter	IEEE Index	ANSYS Index
11	*xx*	1	1
22	*yy*	2	2
33	*zz*	3	3
23 = 32	*yz* = *zy*	4	5
13 = 31	*xz* = *zx*	5	6
12 = 21	*xy* = *yx*	6	4

The constitutive equations link the mechanical *T_p_* and *S_p_* with electric field *E_i_* and electric displacement *D_i_*. Index *p* can assume values from 1 to 6 whereas index *i* can vary from 1 to 3. There are four sets of constitutive equations, depending on which variables are chosen as independent. In our case, the independent variables are strain and electric field. This selection is adopted in the IEEE standard and it is useful in FEM formulations. In a general form, the constitutive equations can be expressed as:

(1) $$T_{p}=T_{p}\left(S,E\right),\;p=1,..,\;6$$

(2) $$D_{i}=D_{i}\left(S,E\right),\;i=1,2,3$$

For low deformations and low electric field, Expressions (1) and (2) can be linearized, giving the classical constitutive equations in the linear range:

(3) $$T_{p}=c_{pq}^{E}S_{q}-e_{kp}E_{k}$$

(4) $$D_{i}=\epsilon_{ik}^{S}E_{k}+e_{iq}S_{q}$$

where *i*, *k* take the values 1, 2, 3 and *p*, *q* take the values 1, 2, 3, 4, 5, 6.

The coefficients $c_{pq}^{E}$, $\epsilon_{ik}^{S}$ and $e_{kp}$ respectively form elastic (*c^E^*), dielectric ($\epsilon^{S}$) and piezoelectric (*e*) matrices. Superindex *E* means constant (short circuit) electric field whereas superindex *S* represents a constant (clamped) mechanical strain. Elastic and dielectric matrices are similar to those used in elasticity and electromagnetic theory for non-piezoelectric materials. The piezoelectric matrix represents the interaction between mechanical and electrical variables. In this case, the partial derivatives are equal and the superindex constant strain or constant electric field is omitted.

(5) $$e_{kp}=-{\left(\frac{\partial T_{p}}{\partial E_{k}}\right)}_{S}={\left(\frac{\partial D_{k}}{\partial S_{p}}\right)}_{E}$$

Due to symmetry and thermodynamic restrictions, the number of independent constants is reduced. In the general case of an anisotropic material there are 21 independent elastic constants, 18 independent piezoelectric constants and six independent dielectric constants. 

After the sinterization process, the piezoelectric ceramic is an aggregate of small crystals. Below the Curie temperature, the crystals form domains or regions with the same polarization. This phenomenon is similar to that observed in ferromagnetism. These domains are randomly oriented and, without external fields, the material does not have global piezoelectric properties, even though the local domains are strongly piezoelectric. To obtain a global piezoelectric behavior, the ceramic must be poled. In this poling process, the ceramic is heated slightly below the Curie temperature and a strong external electric field is applied. The temperature is gradually reduced while maintaining the applied field. At the end, the local domains are statistically aligned in the field direction resulting in a piezoelectric material with polarization in the field direction.

The resulting material presents a symmetry that reduces the number of independent constants in the constitutive equations. In this case, we have a polarization axis and have isotropy in the perpendicular plane. This can be represented by the 6 mm symmetry group in crystals. In this symmetry, there are five independent elastic constants, three independent piezoelectric constants and two independent dielectric constants.

When an external field is applied in the material, part of it is dissipated in an irreversible way. Several authors recognize the existence of dielectric, mechanical and piezoelectric dissipation mechanisms [44]. For the elastic, dielectric and piezoelectric parameters, there exists an accepted definition described in the IEEE standard [42], which considers the piezoelectric materials lossless. In the literature, the losses are introduced by phenomenological parameters such as the *Q* of a resonator, the loss tangent [45] or, in FEM simulations, the Rayleigh parameters [46]. An alternative is introducing the losses using complex numbers in the parameters of the constitutive equations [47]. The use of complex parameters allows adjusting the theoretical to the experimental data in a wide frequency band [19,22]. As a drawback, the number of parameters to identify the model grows from 10 to 20 (10 real parts and 10 imaginary parts). Next, the expressions of the elastic, piezoelectric and dielectric matrices for complex parameters and 6 mm symmetry class are presented.

(6) $$c^{E}=\left[\begin{array}{cccccc} c_{11}+i{\bar{c}}_{11} & c_{12}+i{\bar{c}}_{12} & c_{13}+i{\bar{c}}_{13} & 0 & 0 & 0\\ c_{12}+i{\bar{c}}_{13} & c_{11}+i{\bar{c}}_{11} & c_{13}+i{\bar{c}}_{13} & 0 & 0 & 0\\ c_{13}+i{\bar{c}}_{13} & c_{13}+i{\bar{c}}_{13} & c_{33}+i{\bar{c}}_{33} & 0 & 0 & 0\\ 0 & 0 & 0 & c_{44}+i{\bar{c}}_{44} & 0 & 0\\ 0 & 0 & 0 & 0 & c_{44}+i{\bar{c}}_{44} & 0\\ 0 & 0 & 0 & 0 & 0 & c_{66}+i{\bar{c}}_{66} \end{array}\right]$$

(7) $$e=\left[\begin{array}{cccccc} 0 & 0 & 0 & 0 & e_{15}+i{\bar{e}}_{15} & 0\\ 0 & 0 & 0 & e_{15}+i{\bar{e}}_{15} & 0 & 0\\ e_{31}+i{\bar{e}}_{31} & e_{31}+i{\bar{e}}_{31} & e_{33}+i{\bar{e}}_{33} & 0 & 0 & 0 \end{array}\right]$$

(8) $$\epsilon^{S}=\left[\begin{array}{ccc} \epsilon_{11}+i{\bar{\epsilon }}_{11} & 0 & 0\\ 0 & \epsilon_{11}+i{\bar{\epsilon }}_{11} & 0\\ 0 & 0 & \epsilon_{33}+i{\bar{\epsilon }}_{33} \end{array}\right]$$

where $c_{66}=\left(c_{11}+c_{12}\right)/2$ and ${\bar{c}}_{66}=\left({\bar{c}}_{11}+{\bar{c}}_{12}\right)/2$. The imaginary part of the properties is distinguished by using an over bar and superscripts *E* and *S* are omitted to simplify the notation. Next, those are the matrix considered for the constitutive equations. 

## 3. One-Dimensional Electromechanical Modeling and Impedance Characterization

This section presents some basic concepts about modeling piezoelectric transducers and impedance characterization to obtain the parameters associated to this model. The modeling starts with the simplest deduction of the resonance frequency from the constitutive equations. Then, some one-dimensional classical models are reviewed to show the efforts made to evaluate the constitutive parameters using the impedance curves. Finally, some practical parameters frequently found in the technical literature are introduced. 

### 3.1. One-Dimensional Modeling

**Figure 2:**
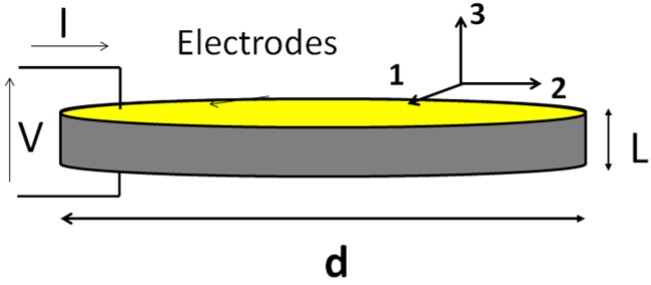
One-dimensional model of a thickness poled piezoelectric ceramic.

A thin slab of piezoelectric ceramic, poled in the normal direction with electrodes at both ends (Figure 2) can be simulated by one-dimensional models [48]. Due to the symmetry of the problem, all vector magnitudes are perpendicular to the ceramic faces and only depend on *z*. 

In this one-dimensional model, the body is only strained in the *z* direction (no shear propagation is considered) and all constitutive parameters are in the polarization direction (*c_33_*, *e*_33_, ε*_33_*). The electrical boundary condition is an open circuit; thus, internal electric field *E* compensates polarization vector *P* producing a null electrical displacement condition *D* = 0. The constitutive Equation (4) for the 3 component can be expressed as:

(9) $$D_{3}=\epsilon_{33}^{S}E_{3}+e_{33}S_{3}=0$$

By substituting the electric field in Equation (3), the stiffness elastic constant *c^D^* is obtained:

(10) $$T_{3}=c_{33}^{E}S_{3}-e_{33}\left(\frac{-e_{33}}{\epsilon_{33}^{S}}\right)S_{3}=c_{33}^{D}S_{3}$$

(11) $$c_{33}^{D}=c_{33}^{E}\left(1+\frac{e_{33}^{2}}{c_{33}^{E}\epsilon_{33}^{S}}\right)$$

Using this elastic constant, the wave Equation for compressional waves can be written as [48]:

(12) $$\frac{\partial^{2}u}{\partial t^{2}}=v_{l}^{2}\frac{\partial^{2}u}{\partial z^{2}}$$

where

(13) $$v_{l}=\sqrt{\frac{c_{33}^{D}}{\rho }}$$

Here, *u* indicates the mechanical displacement, ρ is the density and *v_l_* is the longitudinal velocity of the bulk compressional waves. This wave equation accepts sinusoidal solutions. By imposing both free extremes as the mechanical boundary conditions, the resonance occurs when ceramic thickness *L* is a multiple of half wavelength. The resonance frequency can be expressed as:

(14) $$f_{n}=\frac{n}{2L}\sqrt{\frac{c_{33}^{D}}{\rho }}$$

Due to the symmetry with respect to the central plane, only odd harmonics can be electrically excited (*n* = 1, 3, 5…). 

### 3.2. Electrical Modeling

Resonance in a piezoelectric ceramic involves both electrical and mechanical magnitudes. However, in practice, the ceramic is driven by an electric source and the whole ceramic acts as an equivalent coupled system viewed as the electrical input terminals. This system can be represented by an electric lumped network instead of the electro-mechanical distributed system. 

In a linear electrical network, the voltage and the intensity are related by electrical impedance *Z*. When a sinusoidal voltage is applied to the circuit, the current is also sinusoidal with the same angular frequency *w*. The electrical impedance is a complex function of the angular frequency. Its magnitude is the ratio between the voltage and the current modulus, whereas its phase is the phase shift between current and voltage at each frequency. The real and imaginary parts of the impedance are resistance *R* and reactance *X*, respectively.

(15) Z=R+jX

The inverse of the impedance is also a complex number, electrical admittance *Y*. The real and imaginary parts of the admittance are conductance *G* and susceptance *B*, respectively.

(16) Y=G+jB

The mean power supplied by the voltage source and consumed by the impedance is proportional to conductance *G*. For that reason, the maximum of *G* is associated to the resonance frequency, even in the case of coupling modes. 

**Figure 3:**
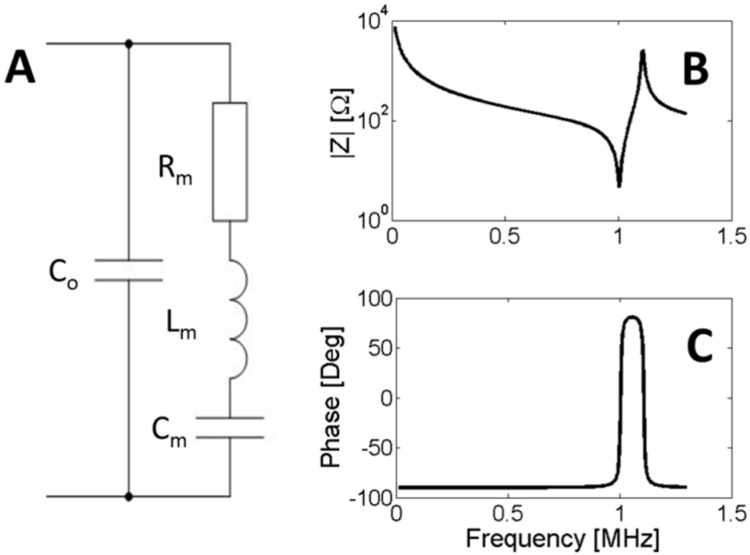
(**A**) Van Dyke equivalent circuit; (**B**) Modulus of the impedance; (**C**) Phase of the impedance. The curves are an example of 1-MHz thickness mode resonance.

In the neighborhood of a resonance, the behavior of a piezoelectric ceramic can be represented by the Van Dyke equivalent circuit [49,50]. This circuit is composed of two shunted branches, one capacitor *C_0_* and the motional branch formed by resistance *R_m_*, inductance *L_m_* and capacitance *C_m_*, see Figure 3.

The use of the Van Dyke equivalent circuit is widely extended in the literature and the basic definitions in the IEEE standard can be interpreted from it. The first step to characterize the ceramic is determining parameters [*C_0_*, *R_m_*, *L_m_*, *C_m_*] from the impedance curve. Experimentally, this can be do using an impedance analyzer that has a predefined set of electrical models to adjust an impedance curve, including the Van Dyke model. The impedance analyzer allows acquiring the electrical impedance as a function of frequency over a desired range. In this case, the parameters can be obtained by using an optimization algorithm to minimize the difference between the model and the experimental data. After that, the material properties can be related to the circuit parameters. A brief description of such relations is presented in the next section. 

The impedance of the Van Dyke circuit can be expressed as:

(17) $$Z=\frac{\left(1-\omega^{2}L_{m}C_{m}\right)+j\omega RC_{m}}{-\omega^{2}R_{m}C_{m}C_{0}+j\omega \left[\left(C_{m}+C_{0}\right)-\omega^{2}L_{m}C_{m}C_{0}\right]}$$

The magnitude and phase of *Z*, given by Equation (17), are shown in Figure 3a,b, respectively. In this topology, we can distinguish two characteristic frequencies, named *f*_1_ and *f*_2_ in the IEEE standard. The first frequency, *f*_1_, is associated with the resonance of the motional branch, also named series resonance. In a lossless material resistor *R* vanishes, and *f*_1_ is defined as the maximum of admittance modulus (*f_m_*), the maximum of conductance (*f_s_*) or zero susceptance (*f_r_*). The frequency of the series resonance can be easily computed as:

(18) $$\omega_{1}=\frac{1}{\sqrt{L_{m}C_{m}}}$$

An external electric generator can drive a piezoelectric ceramic at its resonance frequency *f*_1_. On the other hand, if one capacitor of the Van Dyke circuit is initially charged and the external contacts are open, the circuit oscillates in another frequency. This frequency *f_2_* is the resonance of the parallel branches and can be computed as:

(19) $$\omega_{2}=\frac{1}{\sqrt{L_{m}\frac{C_{0}C_{m}}{C_{m}+C_{0}}}}$$

For the lossless material, *f*_2_ is defined as the maximum of the impedance modulus (*f_n_*), the maximum resistance (*f_p_*) and zero reactance (*f_a_*). As *f*_2_ is the maximum of the impedance modulus, it is also named antiresonance frequency.

**Figure 4:**
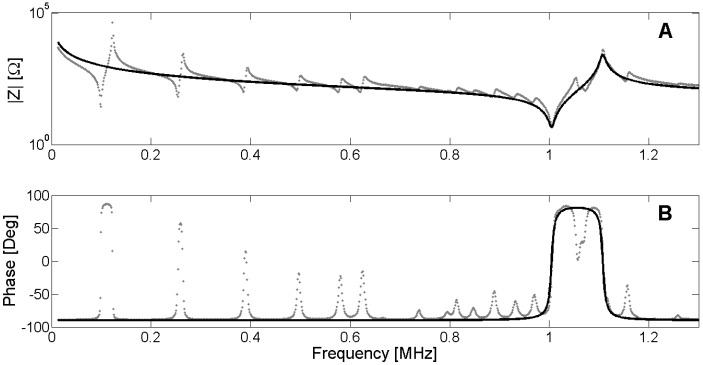
(**A**) Modulus of the electrical impedance; (**B**) Phase of the electrical impedance. Dots are experimental data from 1-mm-thick, 20-mm-diameter PZT Pz27. Black lines are the Van Dyke fitting in the thickness resonance.

In the case of internal losses, the values of *f_m_*, *f_s_* and *f_r_* can differ depending on the value of *R_m_*. The same is valid for *f_n_*, *f_p_* and *f_a_*. To illustrate the determination of the frequencies and the use of the Van Dyke model, a 1-mm-thick 20-mm-diameter PZT Pz27 ceramic is used [1]. Figure 4 shows the fitting of the electrical impedance by using the Nelder-Mead algorithm [16] to minimize the difference between the experimental and the numerical impedances. The Nelder-Mead algorithm is a very easy way to minimize functions, and is available in Matlab by means of the *fminsearch* function.

From the numerical adjustment, the following values are obtained:

(20) $$C_{0}=1.32\;nF;\;C_{m}=0.28\;nF;\;L_{m}=88.5\;\mu H;\;R_{m}=4.56\;\Omega$$

Figure 4 shows that the fitted model is a good approximation in the thickness resonance region. However, the predictions can be affected due to the presence of other resonant modes.

The reactive components in the Van Dyke circuit can be obtained quickly from *f*_1_ using (18), from *f*_2_ using (19) and observing from (17) that, for low frequencies, the impedance is equivalent to the parallel of *C_0_* and *C_m_*. The value of *R_m_* can be obtained from the quality factor *Q* of the circuit:

(21) $$Q=\sqrt{\frac{L_{m}}{R_{m}^{2}C_{m}}}\;≅\frac{f_{1}}{f_{+}-f_{-}}$$

**Figure 5:**
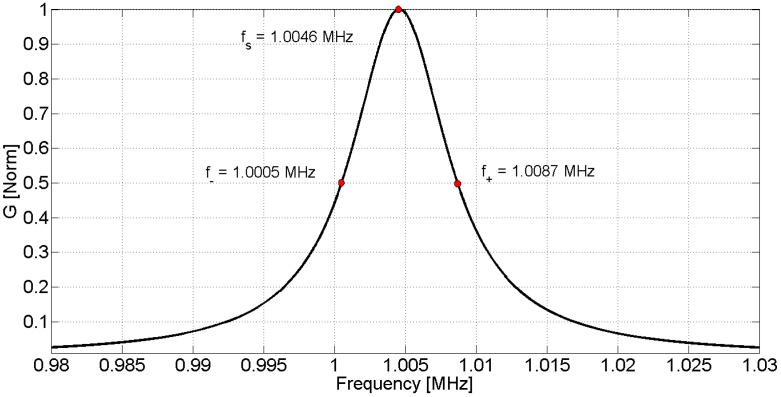
Normalized conductance *G* around the resonance.

Here, *f*_+_ and *f_−_* are the upper and lower half power frequencies, respectively. For high *Q* resonators the expressions in (21) become equal. In practice, for *Q* higher than 10, the expression in (21) differs in less than 0.1%. These values can be used in the optimization algorithm as the initial shot. Figure 5 shows the determination of *f*_+_ and *f*_−_ from the *G* curve.

Using Expression (21), the quality factor is *Q* = 122 for this resonator. In the present example, the internal losses are not negligible and the difference between the characteristic frequencies can be shown.

**Figure 6:**
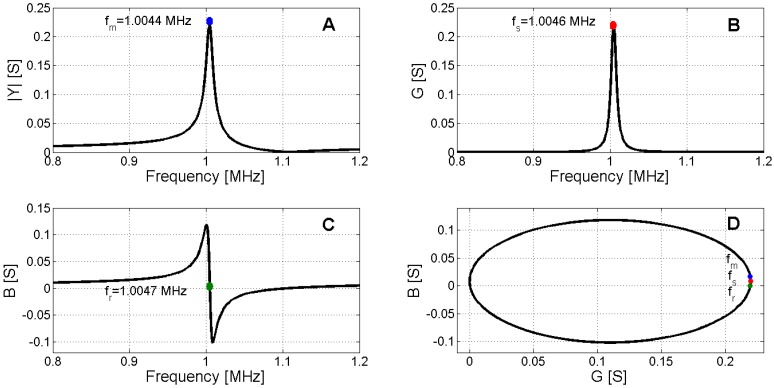
Determination of series resonance. (**A**) Maximum admittance modulus; (**B**) Maximum electric conductance; (**C**) Zero susceptance; (**D**) Polar representation.

**Figure 7:**
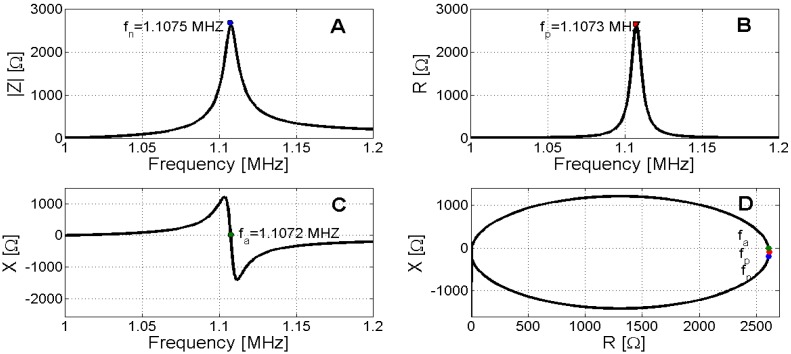
Determination of antiresonace. (**A**) Maximum impedance modulus; (**B**) Maximum electric resistance; (**C**) Zero reactance; (**D**) Polar representation.

Figure 6 and Figure 7 present the determination of *f*_1_ and *f*_2_, respectively.

Manufacturers introduce a set of parameters to compare the performance between different piezoelectric materials. Some examples of parameters that can be directly computed from the impedance data are described below. 

The Dielectric Loss Factor of tan(δ) is computed as the ratio between the real and the imaginary parts of the admittance, usually at 1 kHz:

(22) $$\tan\left(\delta \right)=\frac{G}{\left|B\right|}=\frac{R}{\left|X\right|}$$

In the Pz27 disk used in the present example, tan(δ) = 0.016. 

Frequency Constants *N* are the product of the resonance frequency by the geometrical dimension relevant to this mode. In our example, the relevant dimension is the ceramic thickness *L* of the sample:

(23) $$N_{t}=f_{s}\cdot L=2018\text{ Hz}\cdot m$$

Piezoelectric Coupling Coefficient *k* is the ratio between the mechanical energy stored and the electrical energy supplied by the source in one cycle. There are several values of *k* depending on the geometry and on the polarization axis. In the present example we can compute two values of *k*. The *k_t_* coefficient is associated to the thickness resonance of a disk with diameter much greater than the thickness:

(24) $$k_{t}=\sqrt{\frac{\frac{\pi f_{s}}{2f_{p}}}{\tan\left(\frac{\pi f_{s}}{2f_{p}}\right)}}$$

The other coefficient is the *k_eff_*, or effective coupling coefficient, defined as:

(25) $$k_{eff}=\sqrt{\frac{f_{p}^{2}-f_{s}^{2}}{f_{p}^{2}}}$$

In the present example both coefficients are close to 0.46. 

### 3.3. Electromechanical Model

In the present section, the simplest electromechanical model developed by Mason to link the electrical impedance with the constitutive equation is reviewed [51]. The transducer is simplified to a three-port network, with one electric and two mechanical ports at the electrode surfaces. 

The Mason model relates the mechanical variables (force and velocity) at the external surface to the electrical variables (voltage and current). The piezoelectric ceramic is assumed to be an infinite plate. Using this symmetry, all variables only depend on the thickness direction (3 in our example). In this case, the electrical impedance of the ceramic can be expressed as [48]:

(26) $$Z=\frac{L}{j\omega A\varepsilon_{33}^{s}}\left(1-\frac{2\left(c_{33}^{D}-c_{33}^{E}\right)}{\omega L\sqrt{\rho c_{33}^{D}}}\tan\left(\frac{\omega L}{2}\sqrt{\frac{\rho }{c_{33}^{D}}}\right)\right)$$

where *A* is the area and *L* the thickness of the ceramic.

**Figure 8:**
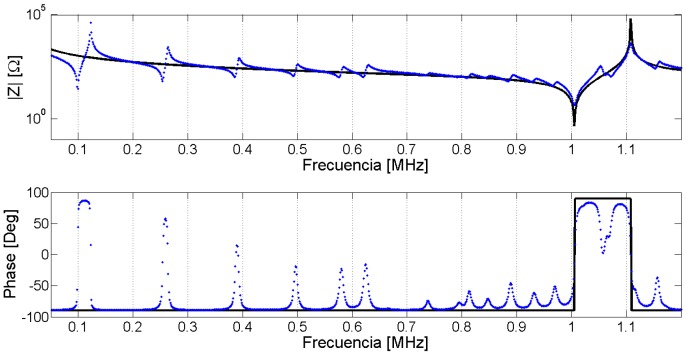
Adjustment of the Mason model of a 2-mm-thick, 20-mm-diameter Pz27 piezoceramic. Blue dots are experimental data whereas continuous black line is the adjusted model.

The constitutive and geometric parameters are the same as those used in Section 3.1. Both surfaces are assumed to be free of external loads. Similar expressions can be obtained for shear polarization or for propagation in other directions using the appropriate symmetry. Following the present example, Figure 8 shows the approximation of the impedance curve using Equation (26). 

From the numerical adjustment, the following values are obtained:

(27) c33E=12.07 1010N/m2;ε33Sε0=850;e33=15.5  C/m2

where ε_0_ is the vacuum permittivity. 

Equation (26) gives the fundamental thickness mode and its harmonics. This expression can be expanded around a resonance and expressed in the Van Dyke format:

(28) $$C_{0}=\frac{A\varepsilon_{33}^{s}}{L}$$

(29) $$C_{m}=\frac{8C_{0}\left(c_{33}^{D}-c_{33}^{E}\right)}{c_{33}^{D}\pi^{2}-8\left(c_{33}^{D}-c_{33}^{E}\right)}$$

(30) $$L_{m}=\frac{c_{33}^{D}\pi^{2}}{\rho L^{2}}\left(1+\frac{8\left(c_{33}^{D}-c_{33}^{E}\right)}{\pi^{2}c_{33}^{E}}\right)$$

More information about the identification of the piezoelectric parameters from the impedance curves can be obtained in the review written by Ballato [52]. 

## 4. Finite Element Method in Piezoelectric Materials

For low deformations, the dynamic behavior of a piezoelectric ceramic can be expressed as a linear system of Partial Differential Equations (PDE). The material structure is divided into elements; each element has a set of nodal points in which the solution is computed. The mechanical or electrical quantities in other points can be interpolated from the nodal solution. The PDEs system representing the Newton and the Gauss law can be expressed as:

(31) $$\rho \ddot{u}=B_{u}^{t}\left(c^{E}B_{u}u-e^{t}B_{\emptyset }\emptyset \right)+f$$

(32) $$B_{\emptyset }^{t}\left(eB_{u}u-\varepsilon^{S}B_{\emptyset }\emptyset \right)=q$$

Here, *u* and φ are the mechanical displacement and the electric potential, respectively, and *f* and *q* are the force and the electric charge density [2,4,6]. Bold letters indicate global vectors at the nodes. *B*_φ_ and *B_u_* are differential operator matrices. Superscript *t* indicates the transpose matrix. Mass density ρ is assumed in the material. The elastic, piezoelectric and dielectric matrices were defined in Section 2.

**Figure 9:**
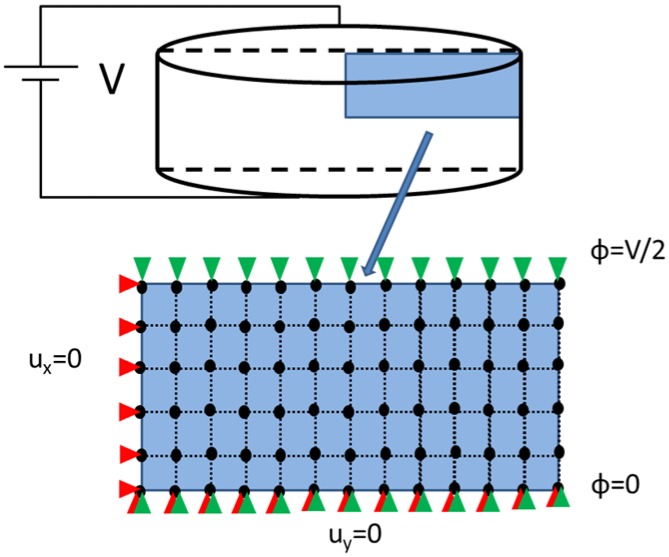
Representation of the symmetry in the piezoelectric disk and boundary conditions in an axisymmetric disk. Black dots indicate nodal positions, red triangles are displacement boundary condition and green triangles are electric potential boundary condition.

For simplicity, the discussion is restricted to two-dimensional axisymmetric elements with linear shape functions. In practice, high order elements are recommended, since they provide a better convergence rate than linear elements. This element allows reducing the 3D model into a 2D model in the case of rotational symmetry. Figure 9 shows the symmetry, the boundary conditions and the mesh grid using 4-node quadrilateral elements. Due to the disk symmetries, only ¼ of the transversal section is used. 

Using 4-node axisymmetric elements and the cylindrical coordinate system (*r,z,*θ), differential operators *B* can be expressed as:

(33) $$B_{u}=\left[\begin{array}{cc} \frac{\partial }{\partial r} & 0\\ 0 & \frac{\partial }{\partial z}\\ \frac{1}{r} & 0\\ \frac{\partial }{\partial z} & \frac{\partial }{\partial r} \end{array}\right]$$

(34) $$B_{\emptyset }=\left[\begin{array}{c} \frac{\partial }{\partial r}\\ \frac{\partial }{\partial z} \end{array}\right]$$

To determine the electrical impedance, a harmonic analysis must be performed. As the system is linear, all the magnitudes change in time as *e^jwt^*. Time is omitted and the equilibrium equation system can be expressed as:

(35) $$\left[\begin{array}{cc} -\omega^{2}M_{uu}+K_{uu} & K_{u\emptyset }\\ K_{u\emptyset }^{t} & -K_{\emptyset \emptyset } \end{array}\right]\left\{\begin{array}{c} U\\ \emptyset \end{array}\right\}=\left\{\begin{array}{c} F\\ Q \end{array}\right\}$$

Where *U*, Φ, *F* and *Q* are the global vectors of mechanical displacements, electric potential, mechanical force, and electric charge, respectively. The capital letters indicate that the values are complex, containing modulus and phase with respect to the reference input, usually the electric potential at the electrode. For example, charge density *q_i_* for the *i*-node can be expressed as:

(36) $$q_{i}\left(t\right)=\left|Q_{i}\right|\cos\left(\omega t+\varphi_{i}\right)=\text{Re}\left\{\left|Q_{i}\right|e^{j\varphi_{i}}e^{j\omega t}\right\}=\text{Re}\left\{Q_{i}e^{j\omega t}\right\}$$

Here *M_uu_*, *K_uu_*, *K_u_*_φ_ and *K*_φφ_ are the mass, elastic, piezoelectric and dielectric global matrices. These matrices can be computed from the constitutive equations by all the elements and by using the appropriate shape functions to describe the nodes [53]. In the case of axisymmetric elements, the matrices of the constitutive equations are reduced. However, all the parameters remain in the model. For this symmetry, the reduced matrices of Equations (6)–(8) are:

(37) $$c^{E}=\left[\begin{array}{cccc} c_{11}+i{\bar{c}}_{11} & c_{12}+i{\bar{c}}_{12} & c_{13}+i{\bar{c}}_{13} & 0\\ c_{12}+i{\bar{c}}_{13} & c_{11}+i{\bar{c}}_{11} & c_{13}+i{\bar{c}}_{13} & 0\\ c_{13}+i{\bar{c}}_{13} & c_{13}+i{\bar{c}}_{13} & c_{33}+i{\bar{c}}_{33} & 0\\ 0 & 0 & 0 & c_{44}+i{\bar{c}}_{44} \end{array}\right]$$

(38) $$e=\left[\begin{array}{cccc} 0 & 0 & 0 & e_{15}+i{\bar{e}}_{15}\\ e_{31}+i{\bar{e}}_{31} & e_{31}+i{\bar{e}}_{31} & e_{33}+i{\bar{e}}_{33} & 0 \end{array}\right]$$

(39) $$\epsilon^{S}=\left[\begin{array}{cc} \epsilon_{11}+i{\bar{\epsilon }}_{11} & 0\\ 0 & \epsilon_{33}+i{\bar{\epsilon }}_{33} \end{array}\right]$$

To determine the electrical impedance, the opposite faces in the polarization direction are metalized. This condition imposes a fixed electric potential for the nodes at the electrodes. The impedance relates the voltage applied to the electrode at frequency *w* with the electric current. The electric current can be computed as the sum of the electric charges at the electrode multiplied by *jw*

(40) $$i\left(t\right)=\frac{d\left(\sum^{\text{​}}q_{i}\right)}{dt}=\frac{d\left(Re\left\{\left|Q_{i}\right|e^{j\varphi_{i}}e^{j\omega t}\right\}\right)}{dt}=Re\left\{j\omega e^{j\omega t}\sum^{\text{​}}\left|Q_{i}\right|e^{j\varphi_{i}}\right\}$$

There are some types of commercial software that implement the FEM simulations [54,55,56]. The user must obtain the elements that allow piezoelectric properties with the appropriate symmetry. A practical issue when selecting the FEM program is the damping model used to introduce the energy losses in the structure. FEM programs use complex parameters, as shown in the constitutive equations, or alternatively use Rayleigh damping parameters α and β to introduce the energy losses. Parameter α is a damping constant associated to the mass matrix of the structure whereas β is a damping constant associated to the elastic matrix. 

To use the FEM in the optimization loop, the finite element software package must generate a data output that can be read automatically; also, the parameters must be changed by the optimization program before the FEM simulation is restarted. 

**Figure 10:**
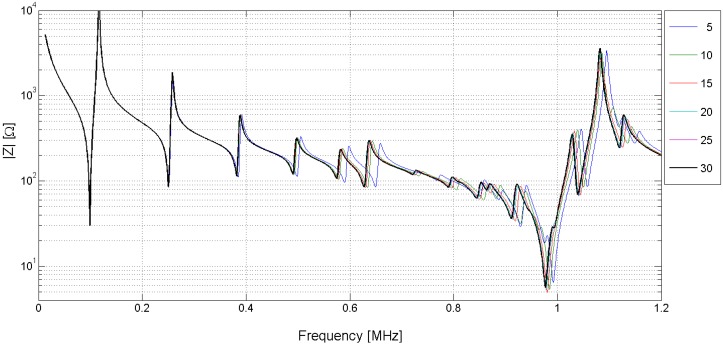
Electrical impedance curves obtained by using different mesh discretizations. The label is the number of elements in the thickness direction. The thick black line is the highest refinement mesh with 30 elements in the thickness.

**Figure 11:**
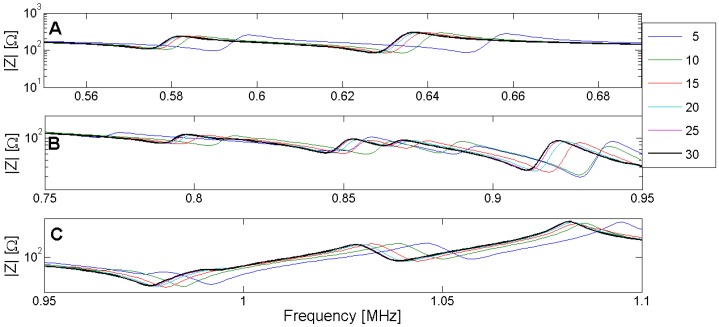
(**A**) Zoom of the convergence in the edge mode. (**B**) Zoom of the convergence in the coupled mode band. (**C**) Zoom of the convergence in the thickness mode.

Another important consideration is the number of elements in the structure to assure the convergence of the FEM results. The difference between simulations made using different element sizes depends on the element type, the frequency and the resonance mode. Figure 10 and Figure 11 show the different solutions for the piezoelectric ceramic Pz27 used as an example, the element shape is four-node square. The solutions are labeled by the number of elements in the thickness; for example, 5 indicates five elements in the thickness corresponding to the 0.4-mm-length element. The convergence of the solution is achieved when the number of elements increases. 

As a final remark, the efficiency of the FEM simulation can be improved by using customized finite element code. This code can be programmed to be coherent with the optimization algorithm and can use strategies, such as adaptive mesh or more elaborated elements [14,19,32]. 

## 5. Finite Element Method (FEM) Optimization Techniques

In a FEM optimization technique, the parameters of the constitutive Equations (3) and (4) are determined by minimizing the difference between experimental data and FEM simulation results. In this work, the experimental data is assumed to be the electrical impedance or any other electrical quantity that can be directly derived from the impedance curve. The use of other physical magnitudes, such as the ultrasonic speed or the velocity distribution at the ceramic surface, has also been reported in the literature [15,27]. In this review, only the characterization based on electrical data is covered, and the measurement of other quantities can be used for validation purposes, as described in the next section. 

**Figure 12:**
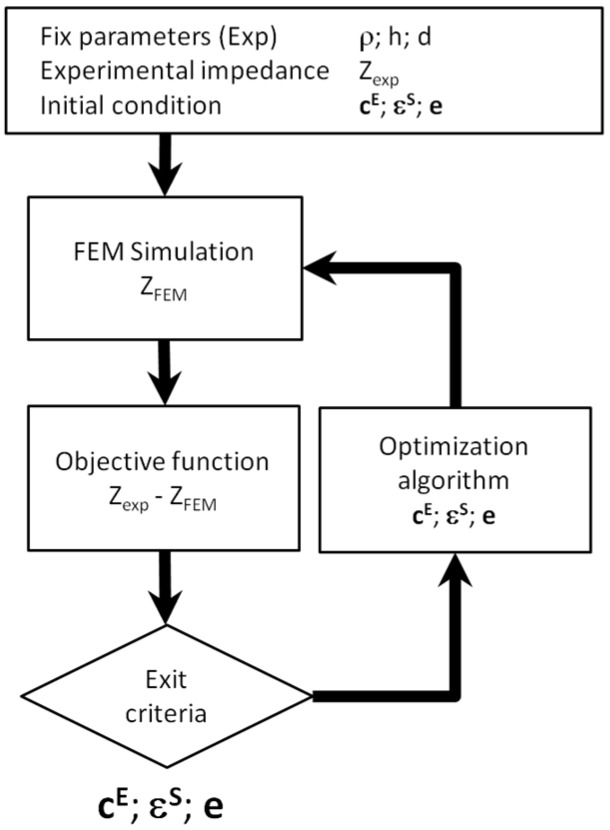
Flowchart of the Finite Element Method (FEM) based optimization.

All the implementations of a numerical optimization share some common steps, as shown in Figure 12:

- Initial conditions: Nonlinear optimization algorithms usually require an initial guess for the material constants.
- FEM computation: The numerical data is obtained from a FEM simulation.
- Objective function: The optimization problem is defined to minimize an objective function.
- Optimization algorithm: Using the value of the objective function the next set of parameters is determined.
- Exit criteria: Usually, the exit criterion is a threshold in the value of the objective function. Alternatively, the number of simulation steps or the difference between two consecutive values in the objective function can be used.

The different FEM techniques used to simulate piezoelectric ceramics are described in Section 4. Next, we expose the main concepts and the different strategies used to solve the other steps in the optimization process.

### 5.1. Problem Statement

In Equation (35), the electric charge can be obtained from the electric potential at the electrodes. In the post-processing of the finite elements, using (40), the electric current and the impedance can be computed. This is a linear problem that relates a set of parameters with a modulus and a phase of the impedance at a given frequency *ϖ* Following the description introduced by Kaltenbacher and Lahmer, the problem can be stated as the mapping of the parameters space *p_par_* to the frequency impedance space *z_par_* [8,12,13]. For the 6 mm symmetry materials, the *p_par_* dimension is 10 for the complex numbers, whereas the dimension of the *z_par_* depends on the number of experimental frequencies *N_freq_* to fit the model. As the parameters are complex numbers, a vector of dimension 10 contains 20 independent real constants. To simplify, no special notation is used to distinguish complex numbers. The parameters to solution operator ℑ give the *N_freq_* complex values of electrical impedance *Z_num_* for an input vector *p_par_*. Here we use notation *Z_num_* to distinguish the numerical solution from experimental values *Z_exp_* measured at the same frequency set.

(41) $$p_{par}=\left[c_{11}^{E},c_{12}^{E},c_{13}^{E},c_{33}^{E},c_{44}^{E},e_{15},e_{31},e_{33},\varepsilon_{11}^{S},\varepsilon_{33}^{S}\right]$$

(42) $$ℑ\left(p_{par}\right)=Z_{num}\;ℑ:{\mathbb{C}}^{N_{par}}\to {\mathbb{C}}^{N_{freq}}$$

**Figure 13:**
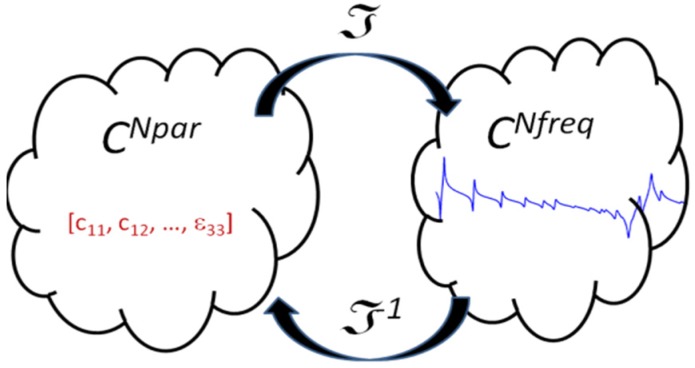
Mapping of the complex parameter space over the impedance complex space.

The objective function for this problem can be stated as the minimization of the error between the experimental and numerical data as shown in Figure 13. The minimization problem can be expressed as:

(43) $$min\left\{\left\|ℑ\left(p_{par}\right)-Z_{exp}\right\|\right\}$$

The inverse problem to obtain vector *p_par_* from a set of impedance values has no general analytical solution. As presented in Section 3, under some particular conditions, the model can be reduced to a one-dimensional problem and some analytical relationships can be obtained. However, for precisely identifying vector *p_par_*, a 3D model must be used, or at least the axisymmetric reduction of the model as shown in Section 4. To solve the problem, the iterative schema of Figure 12 is used.

In Expression (43), we use the norm of the difference for the impedance, however, other physical magnitudes computed from the impedance can be used. For example, conductance *G* and resistance *R* are frequently used. Another issue to define the minimization problem is the *N_freq_* set of experimental points. Here, we can use a narrow or a wide frequency band. One criterion to select the frequency band can be the study of the sensitivity of each parameter. The bandwidth must include resonant modes with appreciable sensitivity for all parameters. This analysis is presented in the next subsection. Once the physical magnitude and the frequency band have been chosen, we can also use weight functions to equalize the different modes in the frequency band. Sometimes, the use of the logarithm of the difference improves the equalization. 

### 5.2. Determination of the Initial Conditions

All FEM optimizations start from an initial set of values for the parameters of the constitutive equations. The exit of the strategy strongly depends on the right choice of the initial conditions. One possibility is to use the literature data as a starting point. These data can be supplied by the manufacturer [1,57] or by scientific papers. Unfortunately, the information is available for few types of piezoelectric materials, but we can obtain a first set by considering a material with similar characteristics. The simulation based on this first set is usually far from the experimental data. There are three different strategies to obtain the initial conditions close to the right values. The most expensive strategy is using brute force by randomly selecting the initial condition in a neighborhood of the first set. This strategy can be useful in the case of low simulation time. 

We can also use a physical approach to determine the initial conditions. The IEEE standard provides some guidelines to obtain different parameters from a set of samples with the adequate geometry. This technique was improved by Sherry and co-authors [58,59,60] and by Alemany and co-authors [61,62], and a complete review describing this methodology was presented by Pardo and Brebol [63].

The third alternative is to apply a sensitivity analysis to estimate how each model parameter affects the resonance frequency of the piezoelectric ceramic vibration modes. Using this information, an approximate algorithm can be constructed to determine the initial condition. This approach was introduced by the authors for the real and imaginary parts of the model and will be detailed as follows [18,19].

**Figure 14:**
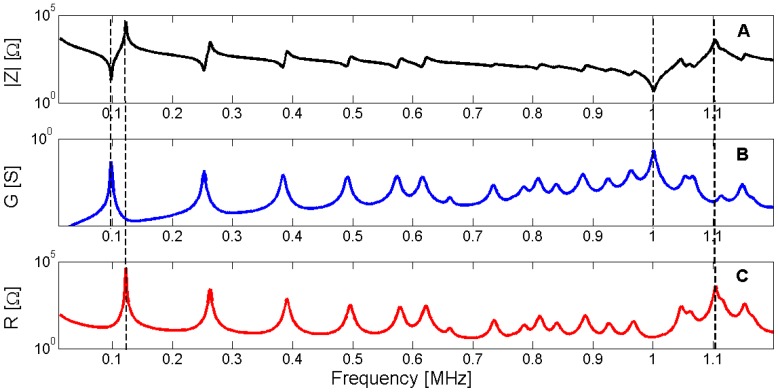
(**A**) Modulus of the electrical impedance for Pz27, 20 mm diameter and 2 mm thickness; (**B**) Conductance *G*; (**C**) Resistance *R*. Dashed lines show the coincidence of the resonance and the antiresonance frequencies with the maximum of *G* and *R*, respectively.

As verified in Section 3.2, the resonance frequency occurs at the maximum of the *G* curve whereas the antiresonance frequency occurs at the maximum of the *R* curve. Figure 14 shows the electrical impedance, conductance *G* and resistance *R* for the same piezoelectric ceramic used in Section 3. To show the determination of the resonance and antiresonance frequencies using the *G* and *R* curves, the first radial mode and the thickness mode are highlighted.

For this reason, the sensitivity analysis is performed by representing the evolution of the maximum of *G* and *R* curves when a given parameter is changed in the model. Next, Figure 15, Figure 16, Figure 17, Figure 18, Figure 19, Figure 20, Figure 21, Figure 22, Figure 23 and Figure 24 show the sensitivity analysis when each parameter is changed from −20% to +20% of its initial values.

**Figure 15:**
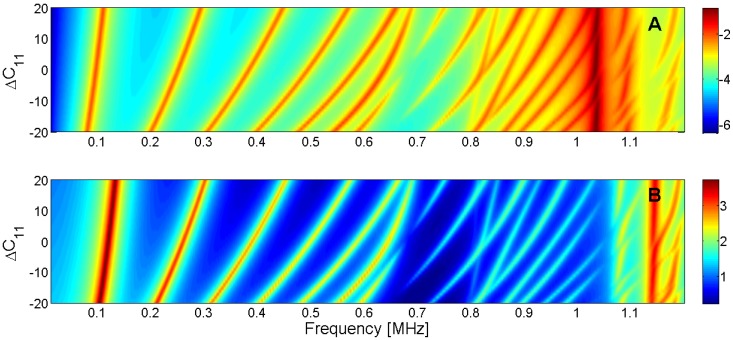
Sensitivity analysis for the real part of *c*_11_ (**A**) Maximum of electric conductance *G*; (**B**) Maximum of resistance *R*.

**Figure 16:**
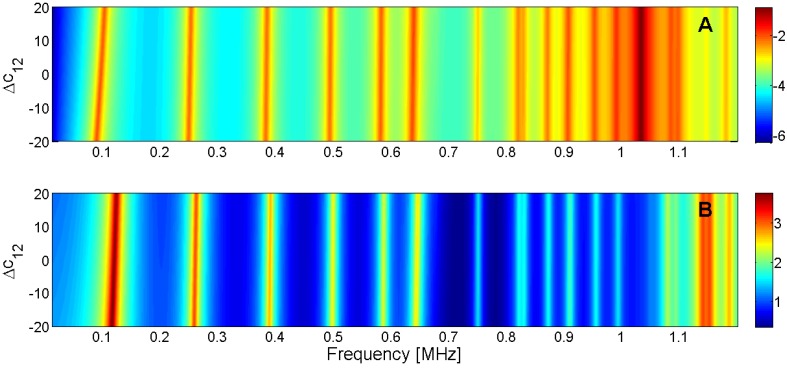
Sensitivity analysis for the real part of *c*_12_ (**A**) Maximum of electric conductance *G*; (**B**) Maximum of resistance *R*.

**Figure 17:**
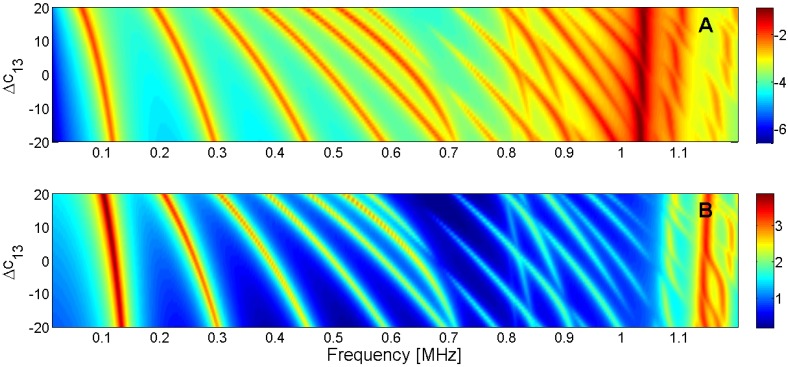
Sensitivity analysis for the real part of *c*_13_ (**A**) Maximum of electric conductance *G*; (**B**) Maximum of resistance *R*.

**Figure 18:**
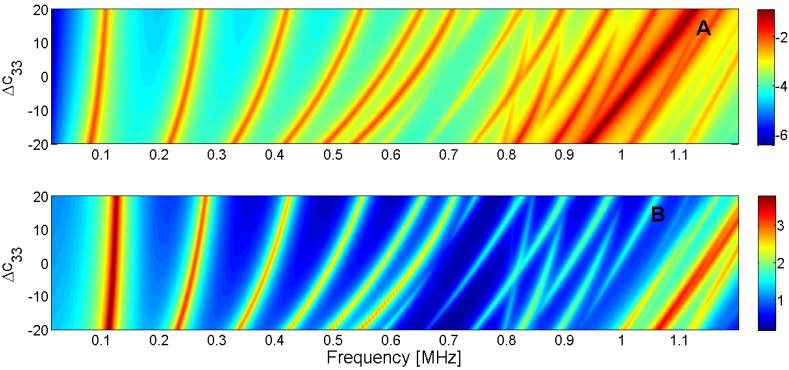
Sensitivity analysis for the real part of *c*_33_ (**A**) Maximum of electric conductance *G*; (**B**) Maximum of resistance *R*.

**Figure 19:**
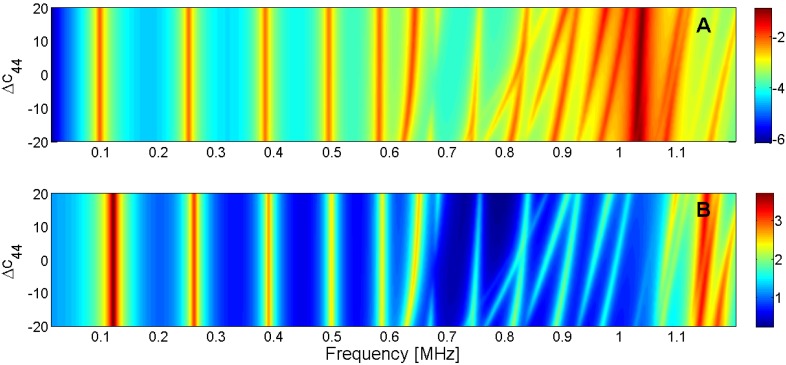
Sensitivity analysis for the real part of *c*_44_ (**A**) Maximum of electric conductance *G*; (**B**) Maximum of resistance *R*.

**Figure 20:**
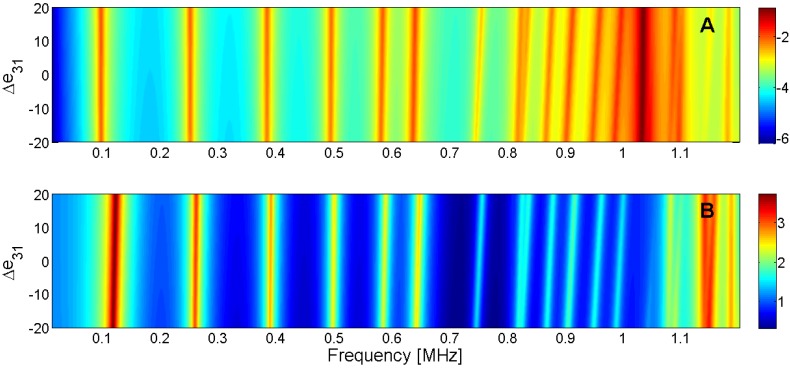
Sensitivity analysis for the real part of *e*_31_ (**A**) Maximum of electric conductance *G*; (**B**) Maximum of resistance *R*.

**Figure 21:**
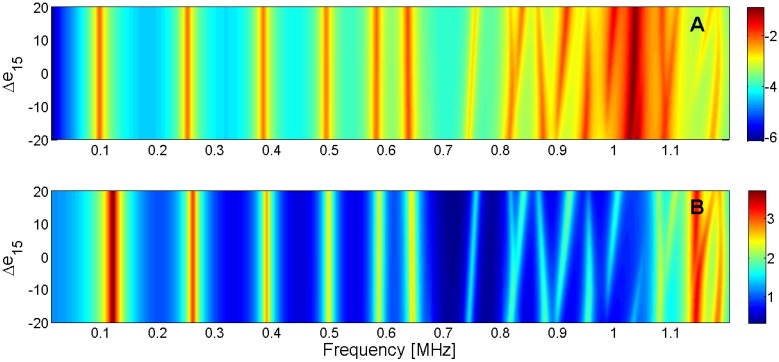
Sensitivity analysis for the real part of *e*_15_ (**A**) Maximum of electric conductance *G*; (**B**) Maximum of resistance *R*.

**Figure 22:**
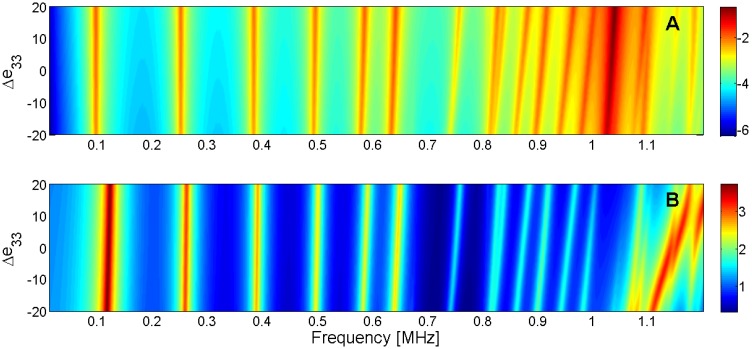
Sensitivity analysis for the real part of *e*_33_ (**A**) Maximum of electric conductance *G*; (**B**) Maximum of resistance *R*.

**Figure 23:**
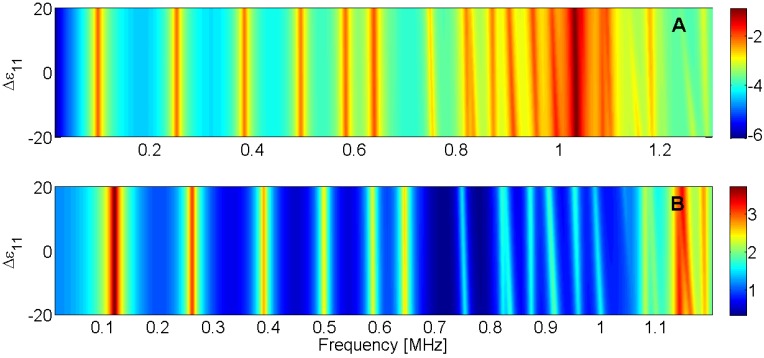
Sensitivity analysis for the real part of ε_11_ (**A**) Maximum of electric conductance *G*; (**B**) Maximum of resistance *R*.

**Figure 24:**
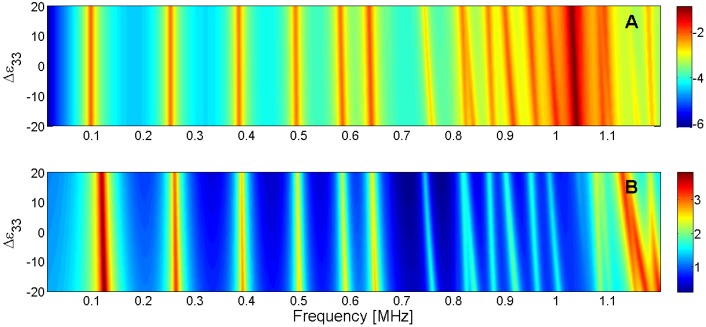
Sensitivity analysis for the real part of ε_33_ (**A**) Maximum of the electric conductance *G*; (**B**) Maximum of the resistance *R*.

**Figure 25:**
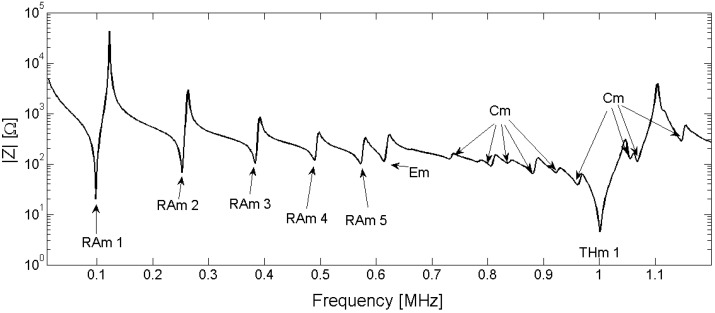
Resonance modes in a Pz27 (20-mm-diameter and 2-mm-thick piezoelectric ceramic). *Ram* is the radial mode, *Em* is the edge mode, *Cm* is coupled mode between radial and thickness resonance, and *THm* is the thickness resonance.

**Figure 26:**
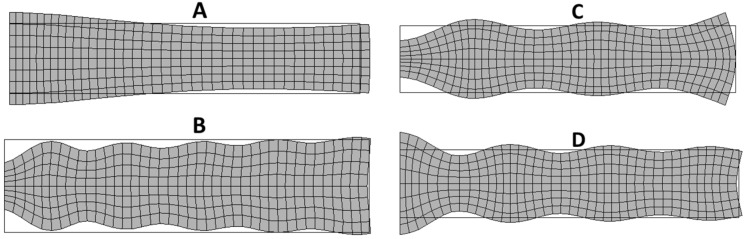
Vibration modes of a piezoelectric disk: (**A**) Second radial mode (*RAm* 2); (**B**) Edge mode (*Em*); (**C**) Coupled mode (*Cm*) between thickness and edge modes; (**D**) Thickness mode (*THm* 1).

To analyze the results, the resonant modes are classified by their origin. In the frequency band below the first thickness mode, we can distinguish four different modes: the radial modes *RAm*, the edge mode *Em*, the coupled modes *Cm* and the thickness mode *THm*. Figure 25 present presents the electrical impedance curve of a piezoelectric disk, with indication of the vibration modes. The vibration patterns of the second radial mode (*Ram* 2), the edge mode (*Em*), the coupled mode (*Cm*) between thickness and edge modes, and thickness mode (*THm* 1) are illustrated in Figure 26. 

The results of the sensitivity analysis are summarized in Table 2. The results are valid for the current example of Pz27, however similar results can be obtained by performing a FEM study of the sensitivity for each parameter in a given thickness/diameter relation. The effect of the variation of each parameter is classified as low, high or no influence. In the case of an appreciable influence, the slope of the curve is included in the table.

**Table 2:** Results of the sensitivity analysis.

Parameter	Radial Mode	Edge Mode	Coupled Mode	Thickness Mode
*c_11_*	High, +slope	High, +slope	High, +slope	No influence
*c_12_*	Low, +slope	No influence	No Influence	No influence
*c_13_*	High, −slope	High, −slope	High, −slope	No influence
*c_33_*	High, +slope	High, +slope	High, +slope	High, +slope
*c_44_*	No influence	High, +slope	High, +slope	Low, +slope
*e_13_*	Low, +slope	Low, +slope	Low, +slope	No influence
*e_15_*	No influence	No influence	High, ±slope	No influence
*e_33_*	Low, +slope	Low, −slope	Low, +slope	High, +slope
ε*_11_*	No influence	No influence	Low, −slope	No influence
ε*_33_*	Low, −slope	Low, −slope	Low, −slope	High, −slope

Similar results can be obtained by analyzing the imaginary part of the model [19]. However, the imaginary part is not critical in the convergence of the algorithm. Here, we are only giving the initial condition for the optimization algorithm. From our experience, for the imaginary part of the model a first approximation using 1% of the real part can be used to start the optimization.

**Figure 27:**
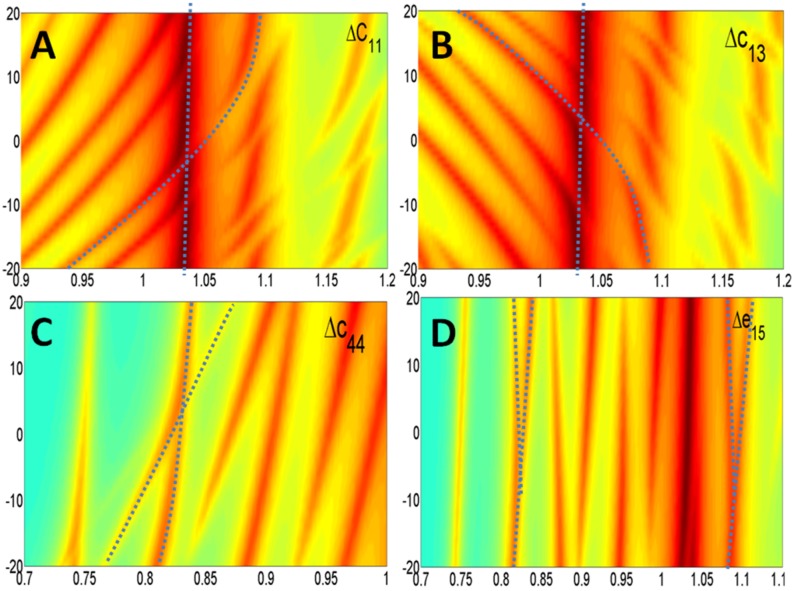
Crossover of resonant modes. (**A**) Cross of a coupled mode over thickness mode in *c*_11_; (**B**) Cross of a coupled mode over thickness mode in *c*_13_; (**C**) Cross of a coupled mode over *E* mode in *c*_44_; (**D**) Cross of coupled modes in *c*_15_.

Real and imaginary parts of the model differently influence the optimization algorithm convergence. The imaginary part takes into account the energy losses without an appreciable change in the resonant frequencies. Additionally, the amplitude of each mode changes smoothly when the imaginary part is increased or decreased. On the other hand, the real part determines the frequency of the resonant modes. A major problem occurs when two lines intersect in the sensitivity diagram. This situation is shown in Figure 27.

One alternative to solve this problem is to implement a preliminary algorithm to approach the initial condition. This algorithm gives a first solution for the more sensitive parameters. An implementation example can be found in [18]. 

### 5.3. Optimization Algorithm

There are many strategies to implement an optimization algorithm able to determine the material parameters. Several authors reported the details for implementing such algorithms [8,12,14,15,32]. The minimization problem to be solved is defined in Section 5.1. The implementation and the validation of efficient algorithms is still an active research field [12]. Additionally, several factors make the practical problem more difficult than the ones stated by the analytical models. Analytical modeling assumes a homogeneous, 6 mm symmetry class material without eccentricity or lack of parallelism. Ideally, the input data have no noise that compromise the solution, the measurement system is absolutely perfect, and so on. In this sense, the results must be validated experimentally using an independent data set. This important question is addressed in the next Section.

It is not our intention to make a detailed discussion about the different algorithms used to solve this problem. Here, we only present the intuitive idea behind some algorithms found in literature. First, we can divide the algorithms into two categories: algorithms that use the gradient approach and algorithms that use the simplex minimization approach. 

The Newton methods are a set of gradient-based method that uses the linear approximation of the functional to find its minimum. We are looking for parameter vector ${\bar{p}}_{par}$ that minimizes the Expression (43). Starting from an initial vector $p_{par}^{0}$ the solution is approximated step by step. At step *k*, the parameter vector is $p_{par}^{k}$ and the next value of the functional can be approximated by:

(44) $$ℑ\left(p_{par}^{k+1}\right)=ℑ\left(p_{par}^{k}\right)+\nabla \left(ℑ\left(p_{par}^{k}\right)\right)\Delta p_{par}^{k}$$

Here $\Delta p_{par}^{k}$ is the increment in the parameter vector at step *k* and gradient ∇ is evaluated at position $p_{par}^{k}$ The gradient can be computed numerically as a difference between two adjacent solutions by running another FEM simulation following the same direction as in the previous step. A more efficient computation can be obtained from the PDE equations with the appropriate analytical expressions [12,32]. Then, the next value of the parameter is:

(45) $$p_{par}^{k+1}=p_{par}^{k}+\Delta p_{par}^{k}$$

where the increment $\Delta p_{par}^{k}$ is computed from the error between the solution and the experimental data:

(46) $$\nabla \left(ℑ\left(p_{par}^{k}\right)\right)\Delta p_{par}^{k}=Z_{exp}-ℑ\left(p_{par}^{k}\right)$$

On the other hand, the Nelder-Mead simplex method [16] is an unconstrained minimization algorithm that does not use the gradient of the functional. This method is widely used to fit models to experimental data. One popular implementation of this algorithm is the *fminsearch* Matlab^®^ (MathWorks, Natick, MA, USA) function. To perform a Nelder-Mead optimization, first a simplex polygon is constructed in the parameters space with one more vertex than the number of the space dimension, for example, in the case of 10 parameters, the simplex polygon has 11 vertices. Each vertex parameter vector named $p_{par,i}$ with *i* from 1 to 11. The objective function is evaluated at each vertex; from this set, the highest and the lowest values of $ℑ\left(p_{par,i}\right)$ are selected

(47) $$ℑ\left(p_{par,low}\right)=min\left\{ℑ\left(p_{par,i}\right)\right\}$$

(48) $$ℑ\left(p_{par,high}\right)=max\left\{ℑ\left(p_{par,i}\right)\right\}$$

As we want to evolve to a minimum, $ℑ\left(p_{par,high}\right)$ is excluded and the centroid of the resulting subset $\langle p_{par,i\neq high}\rangle$ is computed. From this, a straight line, including $\langle p_{par,i\neq high}\rangle$ and $p_{par,high}$ is constructed. A new simplex is constructed by adding a new vertex $p_{par,new}$ reflected from the $\langle p_{par,i\neq high}\rangle$ over the straight line:

(49) $$p_{par,new}=\;\langle p_{par,i\neq high}\rangle +\alpha \left(\langle p_{par,i\neq high}\rangle -p_{par,high}\right)$$

Here α is named the reflection coefficient. By taking α > 0, the $p_{par,new}$ vector belongs to the opposite subspace. To make the search more efficient in the Nelder-Mead implementation, two additional operations are defined: expansion and contraction. In the case of an objective function $ℑ\left(p_{par,new}\right)$ lower than $ℑ\left(p_{par,low}\right)$, the algorithm goes in the right direction and parameter α is increased. In the opposite case, where $ℑ\left(p_{par,new}\right)$ is higher than $ℑ\left(p_{par,high}\right)$, the algorithm jumps too much and the parameter α is decreased. Following these simple rules, the algorithm converges to a local minimum. 

## 6. Validation Methods

**Figure 28:**
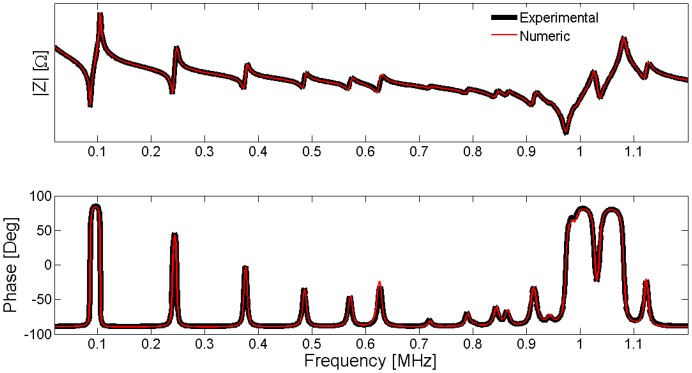
Validation of the results using electrical impedance curves.

After the optimization process, we obtain the complete set of parameters *p_par_* that minimizes the Expression (43). The agreement between the experimental and numerical electrical impedance curves is the first validation. This question seems to be obvious because it is the objective function to be minimized. However, there is no common criterion between researchers to compare the quality of the results. To make an objective comparison between the different methods, the parameters must be identified in the same sample and using the same experimental input data. The frequency data set for evaluation must include resonant modes with appreciable sensitivity for all parameters. As this benchmark does not exist, you can qualitatively determine if all modes are well represented by the FEM simulation. All the evaluations in this section are made in the Pz27, diameter 20 mm, thickness 2 mm, used along this work. The frequency vector has 1000 equally spaced points starting from 13 kHz and ending at 1.3 MHz. Figure 28 presents the modulus and phase of the electrical impedance obtained after the optimized material properties.

The FEM methodology achieves a good approximation between the numerical and experimental curves, indicating that the optimized material properties reproduce the dynamic behavior of the piezoelectric ceramic. However, the validation is made using the same experimental data used to fit the model. The validation of the results using a different experimental data set is highly desirable. One option is using the electrical impedance outside the frequency range used to fit the numerical and experimental data. For example, one can use a frequency band around the third harmonic of the thickness mode taking into account the number of elements to assure the convergence. 

**Figure 29:**
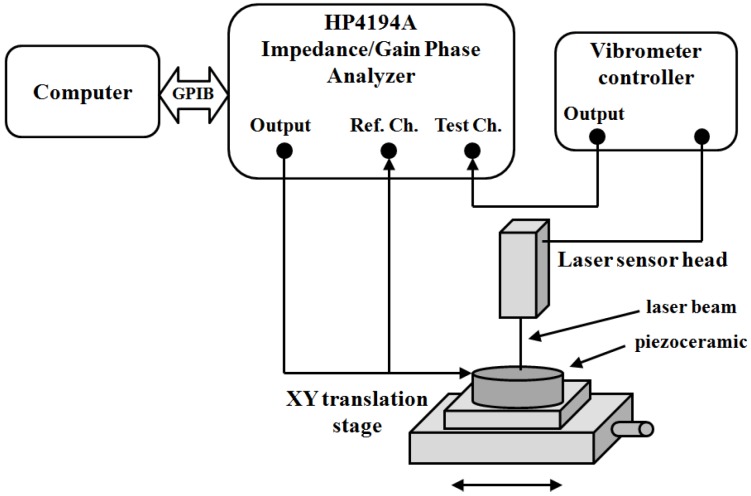
Experimental set up for measuring the vibration pattern of the piezoelectric ceramic surfaces as a function of frequency [18].

The other option is using a mechanical magnitude to validate the methodology in an independent experiment. Several techniques can be used. Lin and Ma measure and compare the FEM results by analyzing the displacement pattern on the surface of a piezoelectric disk using speckle pattern interferometry [64]. Wang and Cao use the phase velocities in a piezoelectric sample to determine the full set of the material parameters [65], the same technique can be used to validate the model adjusted by the impedance data. Fialka and Benes perform a comparative analysis between the results of one-dimensional models in a set of different samples, quasi static measurements and the use of optical interferometry [66]. Here we describe the results of the optical interferometer validation in more detail. Figure 29 shows the experimental assembly to perform the vibration analysis using a single-point Laser Doppler Vibrometer (OFV-534 Sensor Head with an OFV-5000 controller, Polytec GmbH, Waldbronn, Germany). 

**Figure 30:**
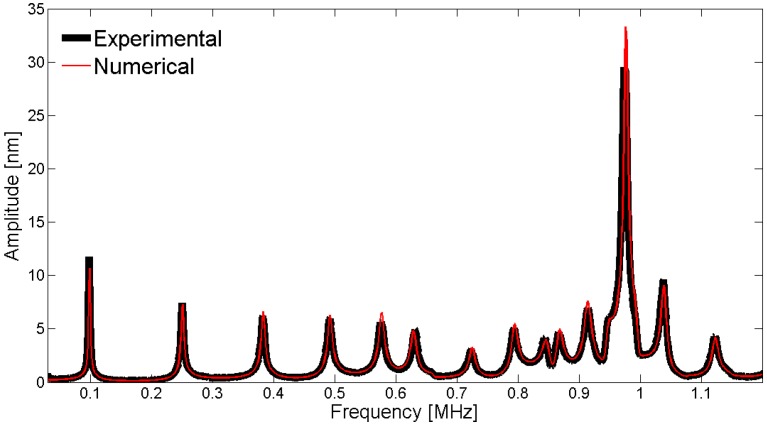
Comparison between the laser vibrometer measurements and the FEM results for the mechanical displacement as a function of frequency at the center point of the piezoelectric disk.

To compare the experimental displacement measurements with the numerical displacements obtained by the adjusted model, we can use a fixed point or scan over a line on the surface. Figure 30 shows the results of the validation using the center point on the electrode surface of the piezoelectric disk. The measured displacements with the interferometer are directly compared to the FEM results without normalization.

**Figure 31:**
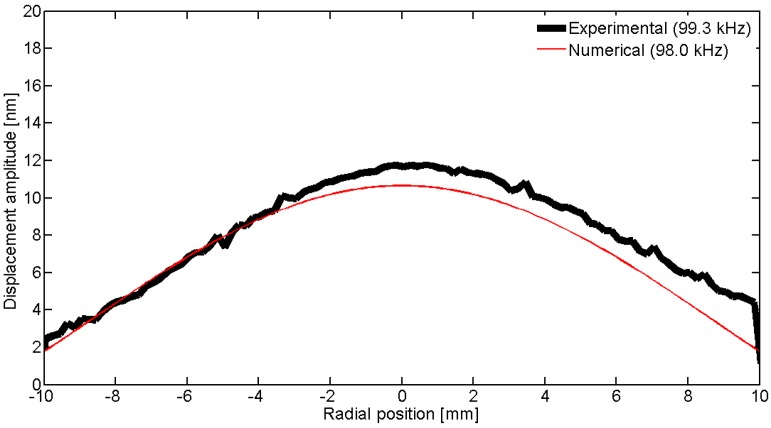
Comparison between the laser vibrometer measurements and the FEM results over a diameter for the first radial mode *RAm1*.

**Figure 32:**
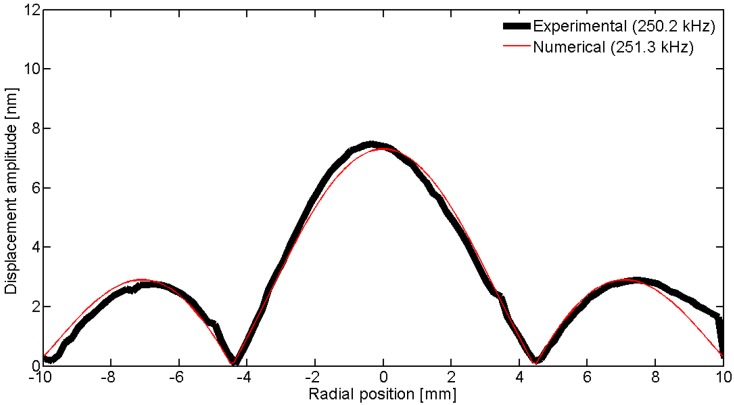
Comparison between the laser vibrometer measurements and the FEM results over a diameter for the second radial mode *Ram2*.

**Figure 33:**
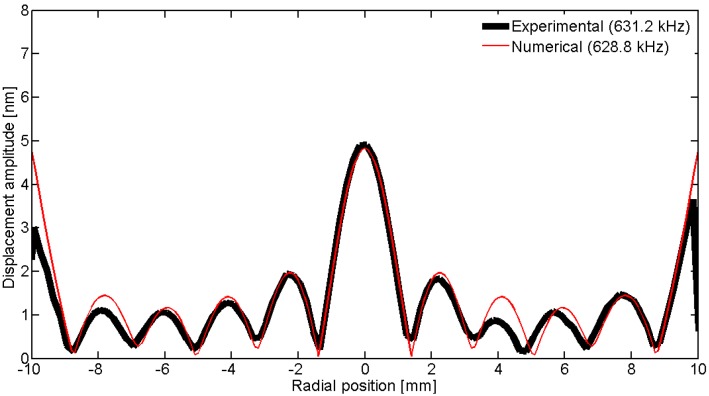
Comparison between the laser vibrometer measurements and the FEM results over a diameter for edge mode *Em*.

**Figure 34:**
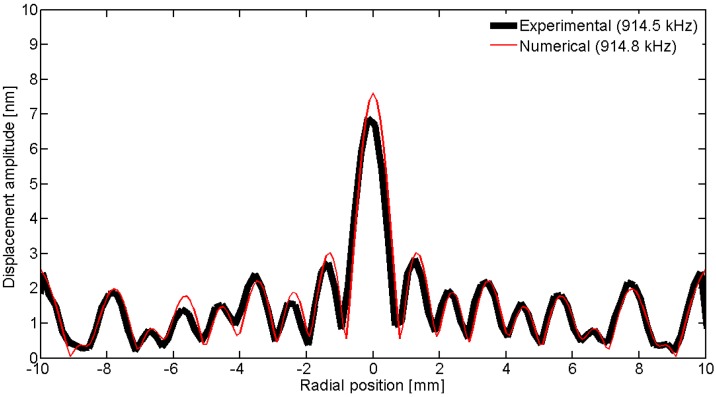
Comparison between the laser vibrometer measurements and the FEM results over a diameter for coupled mode *Cm* at 915 kHz.

**Figure 35:**
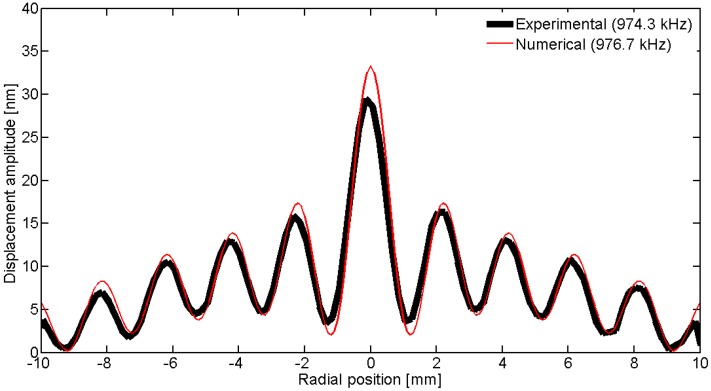
Comparison between the laser vibrometer measurements and the FEM results over a diameter for thickness mode *THm1*.

To observe the spatial pattern of the vibration, we can scan over a line; in this geometry, the natural choice is to use a diameter. Figure 31, Figure 32, Figure 33, Figure 34 and Figure 35 show the comparison over a diameter for displacement. The reference for the modes at the impedance curve is in Figure 29.

**Figure 36:**
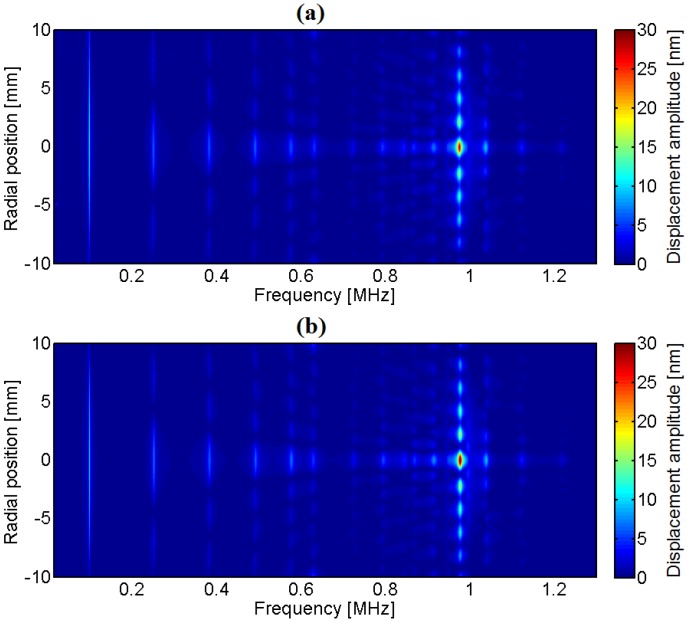
Surface displacement profile along the radial direction as a function of frequency for a 2-mm-thick, 20-mm-diameter Pz27 disk: (**a**) Experimental; (**b**) Numerical [18].

Finally, the comparison in a color map is shown in Figure 36. The horizontal axis is the frequency vector and the vertical axis is the spatial position. The displacement profiles of Figure 31, Figure 32, Figure 33, Figure 34 and Figure 35 correspond to vertical lines in the diagram of Figure 36. 

## 7. Conclusions

This paper presents the recent advances in the characterization of piezoelectric ceramics by numerical optimization methods. The basic strategy to find the material properties by numerical methods can be summarized in the following steps: (1) Measurement of the electrical impedance curve of a piezoelectric sample; (2) Application of a numerical method (such as FEM) combined with an optimization algorithm to find the material parameters; and (3) Results validation. One of the main difficulties is that the objective functions present numerous local minima, which causes optimization algorithms to converge to a set of parameters that does not reproduce the experimental impedance curve. One strategy to minimize the influence of local minima consists in selecting an initial guess closer to the final solution. This is particularly useful when dealing with piezoelectric ceramics of the same type, in which the material parameters of one well known characterized piezoceramic can be used as input to the characterization of another. However, this procedure is not trivial when dealing with ceramics of different types. In the latter case, a sensitivity analysis can assist a human operator to select an initial set closer to the final solution. Despite the recent advances, the characterization of piezoelectric materials by numerical methods is still a very challenging problem, and much effort will be required to develop a new numerical technique to obtain the material parameters of piezoelectric samples with no human intervention.
